# Holiday effect on childbirth: A population-based analysis of 21,869,652 birth records, 1979–2018

**DOI:** 10.1371/journal.pone.0296403

**Published:** 2024-02-14

**Authors:** Miho Sassa, Ryo Kinoshita, Yayoi Murano, Hiromichi Shoji, Daisuke Yoneoka

**Affiliations:** 1 Department of Global Health Policy, Graduate School of Medicine, The University of Tokyo, Tokyo, Japan; 2 Center for Surveillance, Immunization, and Epidemiologic Research, National Institute of Infectious Diseases, Tokyo, Japan; 3 Department of Pediatrics and Adolescent Medicine, Juntendo University, Tokyo, Japan; Siddhi Memorial Hospital, NEPAL

## Abstract

Maternity and neonatal services always have to operate 24 hours a day and 7 days a week, and require well preparedness to guarantee safe deliveries for both mothers and babies. However, the evidence of holiday effect from large-scale data is still insufficient from the obstetrics perspective. We analyzed data of over 21 million births in Japan from January 1, 1979, to December 31, 2018. We revealed that the number of births is lower on holidays, and especially among high-risk births such as low birthweight and preterm births. The frequency of high-risk birth has been increasing over the study period, and the variation by the day of week and between holiday and non-holiday have become more prevalent in recent years.

## Introduction

While newborns and mothers cannot choose when to give birth, medical resources are likely scarce during holidays due to several factors including staffing and hospital policy [1]. Such nonuniform resource allocation accelerates holiday effects: disparities and variations on health outcomes between holiday and non-holiday. While holiday effects have been examined against a range of medical conditions [2], evidence from large-scale and long-term population-based data remain insufficient, and the conclusions arising from the holiday effects are still controversial [3, 4]. This study analyzed the longitudinal variations of 21,869,652 birth records in Japan to quantify holiday effects.

## Method

Birth certificate data from 1979 to 2018, which included information on the individual’s birthday, birthweight and gestational age (GA) was obtained from the Ministry of Health, Labour and Welfare, Japan. We excluded multiple or multiparous births. Each individual was categorized into low-birthweight (LBW, birthweight <2,500g), preterm birth (PTB, GA <37 weeks), or term birth (TB, GA ≥37 weeks). Consequently, the following five groups were considered: LBW, PTB, TB, LBW and preterm birth (LBW-PTB), and LBW and term birth (LBW-TB). We defined Holiday as weekends (Saturday and Sunday), national holidays, substitute holidays [5], Golden-week (April 29—May 5), and new-year period (December 29—January 3). Note that Saturdays were defined as non-holiday until March 1988 [6]. We also defined Long Holiday as three or more consecutive holidays. For simplicity, leap days were excluded from this analysis. The data are available in S1 and S2 Tables.

Tukey’s honestly significant difference test was used to test the statistically significant differences between the day of interest after correcting the inflation of type 1 error originated from the multiple comparisons. In addition, the time trend was tested using a (univariate) linear regression with a time covariate. A p-value of less than 0.05 was considered statistically significant. Statistical analysis was performed using R (version 4.3.1).

## Result

**Fig 1:**
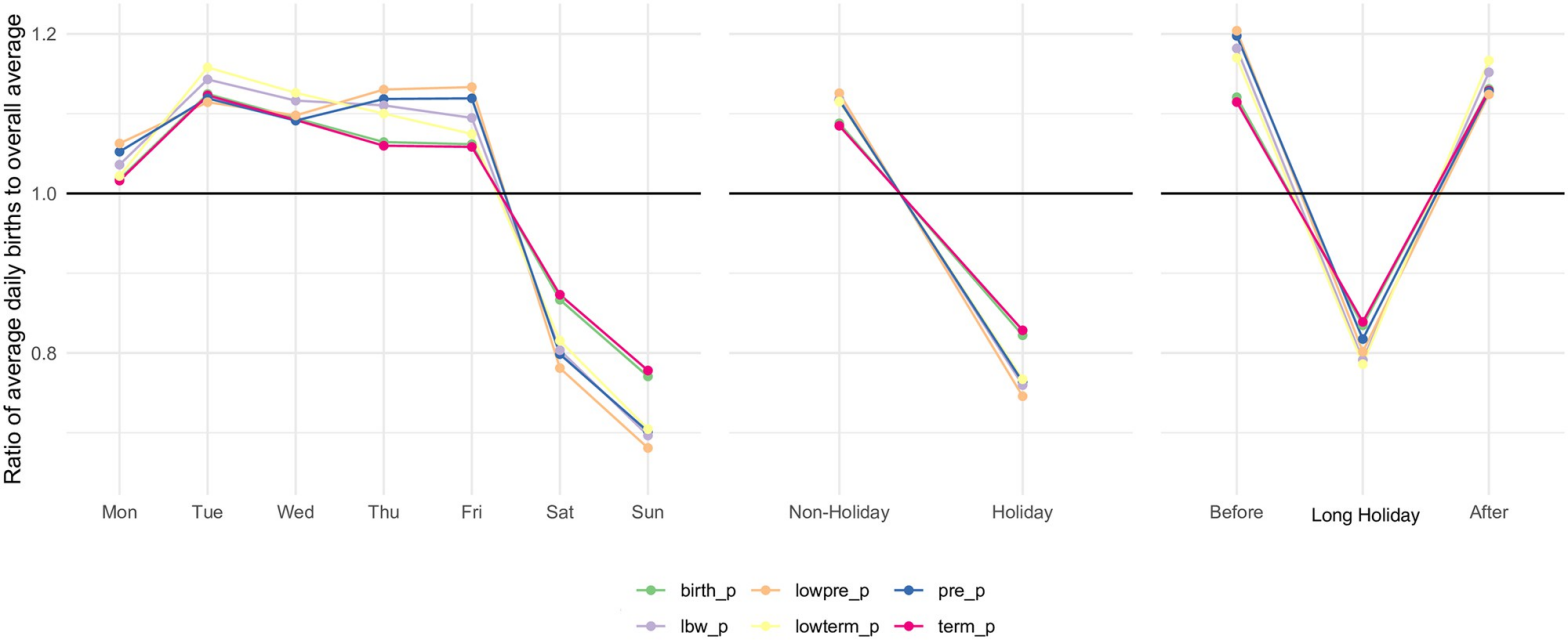
Ratio of daily average number of births to overall average during the recent 10 years. Stratified by day of the week (A), holiday vs non-holiday (B), and long-holiday vs 1–2 days before the holiday vs 1–2 days after the holiday (C). *birth_p: all, lowpre_p: low-birthweight and preterm birth, pre_p: preterm birth, lbw_p: low-birthweight, lowterm_p: low-birthweight and term birth, term_p: term birth.

A total of 21,869,652 (LBW: 7.59%, PTB: 4.13%, LBW-PTB: 2.59%, LBW-TB: 4.99%) births were recorded over the study period. Fig 1 illustrates the ratio of daily average number of births to yearly average during 2009–2018. Clearly, the frequency of all types of births are significantly reduced on weekends (p < 0.01), Holiday (p < 0.01), and Long Holiday (p<0.01). Interestingly, high-risk births such as LBW-PTB are more evidently frequent on Thursday and Friday than low-risk births such as TB (p < 0.01) (Fig 1A). Table 1 shows the detailed results of p-values across all possible combinations after adjustment for the multiple comparisons.

**Table 1:** Estimated p-values in Fig 1 after adjusting for multiple comparisons.

Comparison pairs	All birth	LBW-PTB	PTB	LBW	LBW-TB	TB
Mon vs Fri	<0.001	<0.001	<0.001	<0.001	<0.001	<0.001
Sat vs Fri	<0.001	<0.001	<0.001	<0.001	<0.001	<0.001
Sun vs Fri	<0.001	<0.001	<0.001	<0.001	<0.001	<0.001
Thu vs Fri	0.999	1.000	1.000	0.579	0.157	1.000
Tue vs Fri	<0.001	0.680	1.000	<0.001	<0.001	<0.001
Wed vs Fri	<0.001	0.042	0.086	0.189	<0.001	<0.001
Sat vs Mon	<0.001	<0.001	<0.001	<0.001	<0.001	<0.001
Sun vs Mon	<0.001	<0.001	<0.001	<0.001	<0.001	<0.001
Thu vs Mon	<0.001	<0.001	<0.001	<0.001	<0.001	<0.001
Tue vs Mon	<0.001	<0.001	<0.001	<0.001	<0.001	<0.001
Wed vs Mon	<0.001	0.047	0.002	<0.001	<0.001	<0.001
Sun vs Sat	<0.001	<0.001	<0.001	<0.001	<0.001	<0.001
Thu vs Sat	<0.001	<0.001	<0.001	<0.001	<0.001	<0.001
Tue vs Sat	<0.001	<0.001	<0.001	<0.001	<0.001	<0.001
Wed vs Sat	<0.001	<0.001	<0.001	<0.001	<0.001	<0.001
Thu vs Sun	<0.001	<0.001	<0.001	<0.001	<0.001	<0.001
Tue vs Sun	<0.001	<0.001	<0.001	<0.001	<0.001	<0.001
Wed vs Sun	<0.001	<0.001	<0.001	<0.001	<0.001	<0.001
Tue vs Thu	<0.001	0.833	1.000	0.005	<0.001	<0.001
Wed vs Thu	<0.001	0.087	0.106	0.994	0.140	<0.001
Wed vs Tue	<0.001	0.799	0.087	0.047	0.030	<0.001
Non-Holiday vs Holiday	<0.001	<0.001	<0.001	<0.001	<0.001	<0.001
Weekends vs Weekdays	<0.001	<0.001	<0.001	<0.001	<0.001	<0.001
Long holiday vs Before	<0.001	<0.001	<0.001	<0.001	<0.001	<0.001
After vs Before	0.645	0.420	0.001	0.200	0.001	0.983
After vs Long holiday	<0.001	<0.001	<0.001	<0.001	<0.001	<0.001
Holiday vs Before	<0.001	<0.001	<0.001	<0.001	<0.001	<0.001
After vs Before	<0.001	<0.001	0.311	0.106	0.031	<0.001
After vs Holiday	<0.001	<0.001	<0.001	<0.001	<0.001	<0.001
Weekends vs Before	<0.001	<0.001	<0.001	<0.001	<0.001	<0.001
After vs Before	<0.001	<0.001	<0.001	<0.001	<0.001	<0.001
After vs Weekends	<0.001	<0.001	<0.001	<0.001	<0.001	<0.001

**Fig 2:**
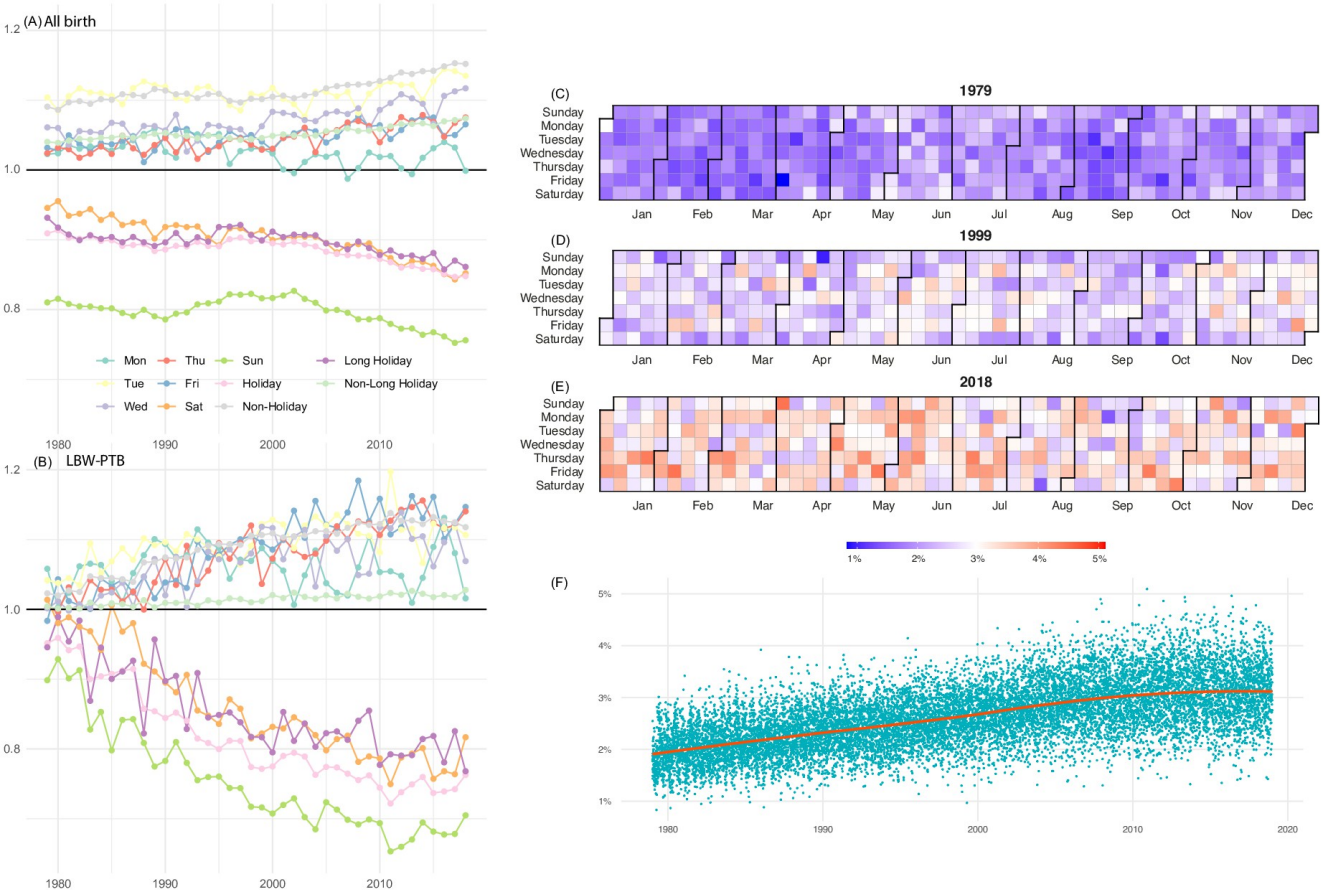
Historical trend of the ratio of daily average number of births to overall average among all birth (A), LBW-PTB (B), calendar of the proportion of LBW-PTB in 1979 (C), 1999 (D), 2018 (E), and the historical trend of the proportion from 1979 to 2018 (F).

Fig 2A and 2B show the historical trend of the ratio defined in Fig 1 among all births and LBW-PTB. In particular, the frequency of LBW-PTB shows a decreasing trend (i.e., less frequent births compared with the average number of births per day) for weekends (p < 0.01), Holiday (p < 0.01), and Long Holiday (p < 0.01).

Fig 2C–2E show the heatmap calendar (Fig 2C–2E) and the historical trend (Fig 2F) of the proportion of LBW-PTB. They imply that high-risk births arising from LBW-PTB has increased over the study period (p < 0.01), and while it was uniformly distributed throughout the year in 1979, recently it has been showing larger daily variation and the difference between high- and low-frequency days becomes enlarged.

## Discussion

This study found that not only the prevalence of high-risk birth has been constantly increasing over the study period, but also recently the weekly cycle and variation between holiday and non-holiday have become more prevalent. The weekly cycle analysis showed that the average daily birth ratio, in comparison to the overall average, starts at a lower level on Monday and gradually increases until Thursday and Friday. However, it sharply decreases on Saturday and Sunday. This pattern could be attributed to the fact that national holidays in Japan often fall on Mondays, which possibly explains the lower ratio observed on Mondays within the weekdays. In addition, our analysis implicates that delivery controlled to prevent adverse effects at Holidays, which are often led by capacity strain (i.e. limited medical resources during Holidays), may be contributing to very low maternal and neonatal mortality in Japan. To avoid high-risk deliveries during Holidays, our finding suggests that many births are controlled to take place 1–2 days before a Holiday, especially on Thursday and Friday among high-risk groups. The limitation of this study includes absence of data on medical intervention and adverse outcomes, which might bias the estimation of holiday effects.

## Conclusion

In conclusion, the large-scale analysis of the births in Japan during 1979–2018 described the increased frequency in birth especially during non-holiday to avoid and prevent adverse effects from birth events during holidays, and such tendency may have contributed to very low maternal mortality and neonatal mortality in Japan. However, there are always mothers who need to give birth during holidays when medical resources are diminished, and holiday deliveries are an inevitable part of routine practice. Systems thinking, including the fair and optimal allocation of medical resources, and all creativity are needed to minimize the risks to those who give birth on holidays and to ensure the safety of all mothers and babies.

## Supporting information

S1 Table Detailed values in ratio of daily average number of births to overall average during the recent 10 years (Fig 1). (DOCX)

S2 Table Historical trend of the ratio of daily average number of births to overall average (Fig 2). (DOCX)
