# Supplementary material for: Holiday effect on childbirth: A population-based analysis of 21,869,652 birth records, 1979–2018
Source: PLoS One. 2024 Feb 14;19(2):e0296403. doi: 10.1371/journal.pone.0296403 (PMC10866518; doi:10.1371/journal.pone.0296403)
Supplement: S1 Table — (DOCX) [file pone.0296403.s001.docx]

|  | birth | term | pre | lbw | lowpre | lowterm |
| --- | --- | --- | --- | --- | --- | --- |
| Mon | 1.018 | 1.016 | 1.052 | 1.036 | 1.063 | 1.022 |
| Tue | 1.125 | 1.123 | 1.119 | 1.143 | 1.114 | 1.158 |
| Wed | 1.094 | 1.092 | 1.091 | 1.116 | 1.098 | 1.126 |
| Thu | 1.064 | 1.060 | 1.118 | 1.110 | 1.130 | 1.100 |
| Fri | 1.062 | 1.058 | 1.119 | 1.095 | 1.133 | 1.075 |
| Sat | 0.867 | 0.873 | 0.798 | 0.803 | 0.781 | 0.815 |
| Sun | 0.771 | 0.778 | 0.701 | 0.697 | 0.681 | 0.705 |
| Non-Holiday | 1.088 | 1.085 | 1.117 | 1.119 | 1.126 | 1.115 |
| Holiday | 0.822 | 0.828 | 0.764 | 0.760 | 0.746 | 0.767 |
| Before | 1.121 | 1.115 | 1.198 | 1.182 | 1.204 | 1.170 |
| Long Holiday | 0.835 | 0.839 | 0.817 | 0.791 | 0.802 | 0.786 |
| After | 1.131 | 1.129 | 1.128 | 1.152 | 1.124 | 1.167 |

S1 Table: Detailed values in ratio of daily average number of births to overall average during the recent 10 years (Figure 1)
