# Supplementary material for: Holiday effect on childbirth: A population-based analysis of 21,869,652 birth records, 1979–2018
Source: PLoS One. 2024 Feb 14;19(2):e0296403. doi: 10.1371/journal.pone.0296403 (PMC10866518; doi:10.1371/journal.pone.0296403)
Supplement: S2 Table — (DOCX) [file pone.0296403.s002.docx]

S2 Table: Historical trend of the ratio of daily average number of births to overall average (Figure 2).

| Year |  | All birth | LBW-PTB |
| --- | --- | --- | --- |
| 1979 | Sun | 0.810 | 0.898 |
| 1979 | Mon | 1.023 | 1.058 |
| 1979 | Tue | 1.104 | 1.042 |
| 1979 | Wed | 1.061 | 1.002 |
| 1979 | Thu | 1.024 | 1.002 |
| 1979 | Fri | 1.032 | 0.983 |
| 1979 | Sat | 0.946 | 1.014 |
| 1979 | Holiday | 0.909 | 0.951 |
| 1979 | Non-Holiday | 1.091 | 1.023 |
| 1979 | Non-Long Holiday | 1.040 | 1.004 |
| 1979 | Long Holiday | 0.932 | 0.946 |
| 1980 | Sun | 0.815 | 0.929 |
| 1980 | Mon | 1.024 | 1.003 |
| 1980 | Tue | 1.085 | 1.038 |
| 1980 | Wed | 1.060 | 1.011 |
| 1980 | Thu | 1.032 | 0.996 |
| 1980 | Fri | 1.028 | 1.043 |
| 1980 | Sat | 0.955 | 0.980 |
| 1980 | Holiday | 0.914 | 0.959 |
| 1980 | Non-Holiday | 1.086 | 1.020 |
| 1980 | Non-Long Holiday | 1.039 | 1.001 |
| 1980 | Long Holiday | 0.918 | 0.989 |
| 1981 | Sun | 0.808 | 0.901 |
| 1981 | Mon | 1.036 | 1.024 |
| 1981 | Tue | 1.106 | 1.045 |
| 1981 | Wed | 1.036 | 0.998 |
| 1981 | Thu | 1.030 | 1.031 |
| 1981 | Fri | 1.050 | 1.012 |
| 1981 | Sat | 0.934 | 0.989 |
| 1981 | Holiday | 0.903 | 0.941 |
| 1981 | Non-Holiday | 1.097 | 1.027 |
| 1981 | Non-Long Holiday | 1.042 | 1.002 |
| 1981 | Long Holiday | 0.908 | 0.954 |
| 1982 | Sun | 0.805 | 0.913 |
| 1982 | Mon | 1.031 | 1.062 |
| 1982 | Tue | 1.117 | 1.035 |
| 1982 | Wed | 1.056 | 1.007 |
| 1982 | Thu | 1.018 | 1.003 |
| 1982 | Fri | 1.037 | 1.006 |
| 1982 | Sat | 0.937 | 0.975 |
| 1982 | Holiday | 0.901 | 0.947 |
| 1982 | Non-Holiday | 1.099 | 1.025 |
| 1982 | Non-Long Holiday | 1.045 | 1.001 |
| 1982 | Long Holiday | 0.900 | 0.984 |
| 1983 | Sun | 0.805 | 0.828 |
| 1983 | Mon | 1.034 | 1.065 |
| 1983 | Tue | 1.111 | 1.095 |
| 1983 | Wed | 1.055 | 1.002 |
| 1983 | Thu | 1.023 | 1.042 |
| 1983 | Fri | 1.028 | 1.001 |
| 1983 | Sat | 0.944 | 0.969 |
| 1983 | Holiday | 0.905 | 0.900 |
| 1983 | Non-Holiday | 1.095 | 1.047 |
| 1983 | Non-Long Holiday | 1.043 | 1.010 |
| 1983 | Long Holiday | 0.907 | 0.869 |
| 1984 | Sun | 0.802 | 0.853 |
| 1984 | Mon | 1.034 | 1.064 |
| 1984 | Tue | 1.110 | 1.052 |
| 1984 | Wed | 1.053 | 1.020 |
| 1984 | Thu | 1.034 | 1.028 |
| 1984 | Fri | 1.038 | 1.044 |
| 1984 | Sat | 0.929 | 0.942 |
| 1984 | Holiday | 0.899 | 0.904 |
| 1984 | Non-Holiday | 1.101 | 1.045 |
| 1984 | Non-Long Holiday | 1.046 | 1.004 |
| 1984 | Long Holiday | 0.901 | 0.945 |
| 1985 | Sun | 0.802 | 0.798 |
| 1985 | Mon | 1.028 | 1.040 |
| 1985 | Tue | 1.106 | 1.069 |
| 1985 | Wed | 1.068 | 1.024 |
| 1985 | Thu | 1.023 | 1.028 |
| 1985 | Fri | 1.037 | 1.034 |
| 1985 | Sat | 0.936 | 1.006 |
| 1985 | Holiday | 0.899 | 0.907 |
| 1985 | Non-Holiday | 1.101 | 1.045 |
| 1985 | Non-Long Holiday | 1.046 | 1.009 |
| 1985 | Long Holiday | 0.904 | 0.900 |
| 1986 | Sun | 0.795 | 0.841 |
| 1986 | Mon | 1.033 | 1.010 |
| 1986 | Tue | 1.094 | 1.087 |
| 1986 | Wed | 1.066 | 1.038 |
| 1986 | Thu | 1.049 | 1.011 |
| 1986 | Fri | 1.040 | 1.042 |
| 1986 | Sat | 0.921 | 0.968 |
| 1986 | Holiday | 0.891 | 0.912 |
| 1986 | Non-Holiday | 1.109 | 1.043 |
| 1986 | Non-Long Holiday | 1.049 | 1.007 |
| 1986 | Long Holiday | 0.897 | 0.911 |
| 1987 | Sun | 0.791 | 0.843 |
| 1987 | Mon | 1.043 | 1.052 |
| 1987 | Tue | 1.118 | 1.071 |
| 1987 | Wed | 1.045 | 1.004 |
| 1987 | Thu | 1.031 | 1.025 |
| 1987 | Fri | 1.048 | 1.024 |
| 1987 | Sat | 0.925 | 0.980 |
| 1987 | Holiday | 0.893 | 0.914 |
| 1987 | Non-Holiday | 1.107 | 1.039 |
| 1987 | Non-Long Holiday | 1.046 | 1.004 |
| 1987 | Long Holiday | 0.904 | 0.926 |
| 1988 | Sun | 0.800 | 0.808 |
| 1988 | Mon | 1.052 | 1.078 |
| 1988 | Tue | 1.127 | 1.102 |
| 1988 | Wed | 1.063 | 1.046 |
| 1988 | Thu | 1.022 | 1.000 |
| 1988 | Fri | 1.011 | 1.047 |
| 1988 | Sat | 0.925 | 0.922 |
| 1988 | Holiday | 0.894 | 0.857 |
| 1988 | Non-Holiday | 1.106 | 1.067 |
| 1988 | Non-Long Holiday | 1.048 | 1.013 |
| 1988 | Long Holiday | 0.896 | 0.822 |
| 1989 | Sun | 0.790 | 0.775 |
| 1989 | Mon | 1.059 | 1.101 |
| 1989 | Tue | 1.122 | 1.092 |
| 1989 | Wed | 1.063 | 1.072 |
| 1989 | Thu | 1.036 | 1.038 |
| 1989 | Fri | 1.028 | 1.016 |
| 1989 | Sat | 0.902 | 0.911 |
| 1989 | Holiday | 0.884 | 0.854 |
| 1989 | Non-Holiday | 1.116 | 1.068 |
| 1989 | Non-Long Holiday | 1.052 | 1.003 |
| 1989 | Long Holiday | 0.891 | 0.957 |
| 1990 | Sun | 0.786 | 0.783 |
| 1990 | Mon | 1.026 | 1.094 |
| 1990 | Tue | 1.119 | 1.096 |
| 1990 | Wed | 1.057 | 1.020 |
| 1990 | Thu | 1.046 | 1.072 |
| 1990 | Fri | 1.048 | 1.038 |
| 1990 | Sat | 0.918 | 0.895 |
| 1990 | Holiday | 0.886 | 0.844 |
| 1990 | Non-Holiday | 1.114 | 1.074 |
| 1990 | Non-Long Holiday | 1.053 | 1.009 |
| 1990 | Long Holiday | 0.896 | 0.897 |
| 1991 | Sun | 0.794 | 0.810 |
| 1991 | Mon | 1.018 | 1.073 |
| 1991 | Tue | 1.104 | 1.083 |
| 1991 | Wed | 1.079 | 1.075 |
| 1991 | Thu | 1.025 | 1.035 |
| 1991 | Fri | 1.058 | 1.041 |
| 1991 | Sat | 0.921 | 0.881 |
| 1991 | Holiday | 0.891 | 0.852 |
| 1991 | Non-Holiday | 1.109 | 1.073 |
| 1991 | Non-Long Holiday | 1.049 | 1.009 |
| 1991 | Long Holiday | 0.910 | 0.905 |
| 1992 | Sun | 0.796 | 0.780 |
| 1992 | Mon | 1.055 | 1.078 |
| 1992 | Tue | 1.100 | 1.108 |
| 1992 | Wed | 1.026 | 1.003 |
| 1992 | Thu | 1.045 | 1.091 |
| 1992 | Fri | 1.059 | 1.031 |
| 1992 | Sat | 0.918 | 0.906 |
| 1992 | Holiday | 0.891 | 0.841 |
| 1992 | Non-Holiday | 1.109 | 1.074 |
| 1992 | Non-Long Holiday | 1.049 | 1.010 |
| 1992 | Long Holiday | 0.894 | 0.828 |
| 1993 | Sun | 0.807 | 0.755 |
| 1993 | Mon | 1.051 | 1.114 |
| 1993 | Tue | 1.117 | 1.100 |
| 1993 | Wed | 1.042 | 1.040 |
| 1993 | Thu | 1.016 | 1.036 |
| 1993 | Fri | 1.047 | 1.097 |
| 1993 | Sat | 0.919 | 0.855 |
| 1993 | Holiday | 0.897 | 0.815 |
| 1993 | Non-Holiday | 1.103 | 1.085 |
| 1993 | Non-Long Holiday | 1.045 | 1.005 |
| 1993 | Long Holiday | 0.904 | 0.909 |
| 1994 | Sun | 0.809 | 0.760 |
| 1994 | Mon | 1.050 | 1.096 |
| 1994 | Tue | 1.120 | 1.088 |
| 1994 | Wed | 1.065 | 1.083 |
| 1994 | Thu | 1.028 | 1.055 |
| 1994 | Fri | 1.026 | 1.074 |
| 1994 | Sat | 0.903 | 0.848 |
| 1994 | Holiday | 0.891 | 0.808 |
| 1994 | Non-Holiday | 1.109 | 1.092 |
| 1994 | Non-Long Holiday | 1.050 | 1.013 |
| 1994 | Long Holiday | 0.898 | 0.845 |
| 1995 | Sun | 0.811 | 0.760 |
| 1995 | Mon | 1.052 | 1.088 |
| 1995 | Tue | 1.109 | 1.076 |
| 1995 | Wed | 1.063 | 1.072 |
| 1995 | Thu | 1.034 | 1.095 |
| 1995 | Fri | 1.039 | 1.079 |
| 1995 | Sat | 0.892 | 0.836 |
| 1995 | Holiday | 0.891 | 0.800 |
| 1995 | Non-Holiday | 1.109 | 1.093 |
| 1995 | Non-Long Holiday | 1.048 | 1.010 |
| 1995 | Long Holiday | 0.918 | 0.849 |
| 1996 | Sun | 0.822 | 0.744 |
| 1996 | Mon | 1.009 | 1.044 |
| 1996 | Tue | 1.093 | 1.089 |
| 1996 | Wed | 1.059 | 1.086 |
| 1996 | Thu | 1.045 | 1.073 |
| 1996 | Fri | 1.056 | 1.092 |
| 1996 | Sat | 0.916 | 0.871 |
| 1996 | Holiday | 0.901 | 0.812 |
| 1996 | Non-Holiday | 1.099 | 1.093 |
| 1996 | Non-Long Holiday | 1.046 | 1.016 |
| 1996 | Long Holiday | 0.919 | 0.852 |
| 1997 | Sun | 0.821 | 0.744 |
| 1997 | Mon | 1.027 | 1.071 |
| 1997 | Tue | 1.085 | 1.064 |
| 1997 | Wed | 1.058 | 1.067 |
| 1997 | Thu | 1.045 | 1.095 |
| 1997 | Fri | 1.046 | 1.100 |
| 1997 | Sat | 0.916 | 0.858 |
| 1997 | Holiday | 0.903 | 0.811 |
| 1997 | Non-Holiday | 1.097 | 1.092 |
| 1997 | Non-Long Holiday | 1.043 | 1.014 |
| 1997 | Long Holiday | 0.921 | 0.838 |
| 1998 | Sun | 0.823 | 0.717 |
| 1998 | Mon | 1.031 | 1.069 |
| 1998 | Tue | 1.109 | 1.091 |
| 1998 | Wed | 1.044 | 1.080 |
| 1998 | Thu | 1.036 | 1.120 |
| 1998 | Fri | 1.050 | 1.105 |
| 1998 | Sat | 0.908 | 0.815 |
| 1998 | Holiday | 0.898 | 0.774 |
| 1998 | Non-Holiday | 1.102 | 1.105 |
| 1998 | Non-Long Holiday | 1.044 | 1.010 |
| 1998 | Long Holiday | 0.907 | 0.817 |
| 1999 | Sun | 0.816 | 0.716 |
| 1999 | Mon | 1.031 | 1.088 |
| 1999 | Tue | 1.107 | 1.122 |
| 1999 | Wed | 1.063 | 1.118 |
| 1999 | Thu | 1.029 | 1.036 |
| 1999 | Fri | 1.041 | 1.096 |
| 1999 | Sat | 0.914 | 0.822 |
| 1999 | Holiday | 0.898 | 0.771 |
| 1999 | Non-Holiday | 1.102 | 1.106 |
| 1999 | Non-Long Holiday | 1.046 | 1.012 |
| 1999 | Long Holiday | 0.899 | 0.817 |
| 2000 | Sun | 0.817 | 0.707 |
| 2000 | Mon | 1.024 | 1.069 |
| 2000 | Tue | 1.118 | 1.128 |
| 2000 | Wed | 1.083 | 1.116 |
| 2000 | Thu | 1.030 | 1.073 |
| 2000 | Fri | 1.029 | 1.086 |
| 2000 | Sat | 0.900 | 0.832 |
| 2000 | Holiday | 0.895 | 0.775 |
| 2000 | Non-Holiday | 1.105 | 1.109 |
| 2000 | Non-Long Holiday | 1.049 | 1.020 |
| 2000 | Long Holiday | 0.912 | 0.795 |
| 2001 | Sun | 0.820 | 0.720 |
| 2001 | Mon | 1.001 | 1.055 |
| 2001 | Tue | 1.102 | 1.122 |
| 2001 | Wed | 1.082 | 1.072 |
| 2001 | Thu | 1.046 | 1.102 |
| 2001 | Fri | 1.046 | 1.099 |
| 2001 | Sat | 0.903 | 0.829 |
| 2001 | Holiday | 0.893 | 0.790 |
| 2001 | Non-Holiday | 1.107 | 1.103 |
| 2001 | Non-Long Holiday | 1.051 | 1.016 |
| 2001 | Long Holiday | 0.904 | 0.853 |
| 2002 | Sun | 0.827 | 0.729 |
| 2002 | Mon | 0.996 | 1.007 |
| 2002 | Tue | 1.094 | 1.087 |
| 2002 | Wed | 1.079 | 1.104 |
| 2002 | Thu | 1.048 | 1.084 |
| 2002 | Fri | 1.053 | 1.141 |
| 2002 | Sat | 0.904 | 0.845 |
| 2002 | Holiday | 0.897 | 0.794 |
| 2002 | Non-Holiday | 1.103 | 1.104 |
| 2002 | Non-Long Holiday | 1.049 | 1.024 |
| 2002 | Long Holiday | 0.908 | 0.811 |
| 2003 | Sun | 0.815 | 0.702 |
| 2003 | Mon | 1.012 | 1.049 |
| 2003 | Tue | 1.077 | 1.120 |
| 2003 | Wed | 1.072 | 1.114 |
| 2003 | Thu | 1.061 | 1.075 |
| 2003 | Fri | 1.059 | 1.102 |
| 2003 | Sat | 0.905 | 0.836 |
| 2003 | Holiday | 0.893 | 0.789 |
| 2003 | Non-Holiday | 1.107 | 1.106 |
| 2003 | Non-Long Holiday | 1.049 | 1.018 |
| 2003 | Long Holiday | 0.907 | 0.836 |
| 2004 | Sun | 0.810 | 0.685 |
| 2004 | Mon | 1.022 | 1.084 |
| 2004 | Tue | 1.112 | 1.133 |
| 2004 | Wed | 1.070 | 1.033 |
| 2004 | Thu | 1.026 | 1.080 |
| 2004 | Fri | 1.055 | 1.155 |
| 2004 | Sat | 0.904 | 0.820 |
| 2004 | Holiday | 0.891 | 0.763 |
| 2004 | Non-Holiday | 1.109 | 1.112 |
| 2004 | Non-Long Holiday | 1.049 | 1.015 |
| 2004 | Long Holiday | 0.906 | 0.803 |
| 2005 | Sun | 0.799 | 0.723 |
| 2005 | Mon | 1.019 | 1.037 |
| 2005 | Tue | 1.112 | 1.120 |
| 2005 | Wed | 1.081 | 1.105 |
| 2005 | Thu | 1.054 | 1.098 |
| 2005 | Fri | 1.039 | 1.121 |
| 2005 | Sat | 0.895 | 0.798 |
| 2005 | Holiday | 0.883 | 0.774 |
| 2005 | Non-Holiday | 1.117 | 1.112 |
| 2005 | Non-Long Holiday | 1.056 | 1.020 |
| 2005 | Long Holiday | 0.895 | 0.823 |
| 2006 | Sun | 0.800 | 0.713 |
| 2006 | Mon | 1.024 | 1.024 |
| 2006 | Tue | 1.106 | 1.135 |
| 2006 | Wed | 1.084 | 1.084 |
| 2006 | Thu | 1.057 | 1.119 |
| 2006 | Fri | 1.048 | 1.117 |
| 2006 | Sat | 0.882 | 0.814 |
| 2006 | Holiday | 0.880 | 0.773 |
| 2006 | Non-Holiday | 1.120 | 1.108 |
| 2006 | Non-Long Holiday | 1.056 | 1.016 |
| 2006 | Long Holiday | 0.893 | 0.825 |
| 2007 | Sun | 0.795 | 0.698 |
| 2007 | Mon | 0.988 | 1.020 |
| 2007 | Tue | 1.112 | 1.122 |
| 2007 | Wed | 1.080 | 1.101 |
| 2007 | Thu | 1.068 | 1.101 |
| 2007 | Fri | 1.064 | 1.139 |
| 2007 | Sat | 0.892 | 0.819 |
| 2007 | Holiday | 0.878 | 0.765 |
| 2007 | Non-Holiday | 1.122 | 1.117 |
| 2007 | Non-Long Holiday | 1.060 | 1.022 |
| 2007 | Long Holiday | 0.886 | 0.813 |
| 2008 | Sun | 0.786 | 0.692 |
| 2008 | Mon | 1.003 | 1.060 |
| 2008 | Tue | 1.082 | 1.108 |
| 2008 | Wed | 1.088 | 1.049 |
| 2008 | Thu | 1.070 | 1.126 |
| 2008 | Fri | 1.077 | 1.184 |
| 2008 | Sat | 0.894 | 0.781 |
| 2008 | Holiday | 0.877 | 0.755 |
| 2008 | Non-Holiday | 1.123 | 1.124 |
| 2008 | Non-Long Holiday | 1.056 | 1.018 |
| 2008 | Long Holiday | 0.898 | 0.840 |
| 2009 | Sun | 0.786 | 0.699 |
| 2009 | Mon | 1.025 | 1.104 |
| 2009 | Tue | 1.109 | 1.105 |
| 2009 | Wed | 1.059 | 1.051 |
| 2009 | Thu | 1.063 | 1.123 |
| 2009 | Fri | 1.063 | 1.126 |
| 2009 | Sat | 0.895 | 0.789 |
| 2009 | Holiday | 0.876 | 0.762 |
| 2009 | Non-Holiday | 1.124 | 1.121 |
| 2009 | Non-Long Holiday | 1.056 | 1.015 |
| 2009 | Long Holiday | 0.888 | 0.855 |
| 2010 | Sun | 0.788 | 0.694 |
| 2010 | Mon | 1.019 | 1.053 |
| 2010 | Tue | 1.122 | 1.081 |
| 2010 | Wed | 1.086 | 1.117 |
| 2010 | Thu | 1.040 | 1.107 |
| 2010 | Fri | 1.064 | 1.158 |
| 2010 | Sat | 0.882 | 0.786 |
| 2010 | Holiday | 0.872 | 0.744 |
| 2010 | Non-Holiday | 1.128 | 1.122 |
| 2010 | Non-Long Holiday | 1.059 | 1.019 |
| 2010 | Long Holiday | 0.878 | 0.777 |
| 2011 | Sun | 0.780 | 0.653 |
| 2011 | Mon | 1.020 | 1.054 |
| 2011 | Tue | 1.127 | 1.197 |
| 2011 | Wed | 1.096 | 1.116 |
| 2011 | Thu | 1.060 | 1.127 |
| 2011 | Fri | 1.044 | 1.107 |
| 2011 | Sat | 0.874 | 0.750 |
| 2011 | Holiday | 0.868 | 0.722 |
| 2011 | Non-Holiday | 1.132 | 1.138 |
| 2011 | Non-Long Holiday | 1.063 | 1.023 |
| 2011 | Long Holiday | 0.885 | 0.792 |
| 2012 | Sun | 0.773 | 0.660 |
| 2012 | Mon | 1.002 | 1.048 |
| 2012 | Tue | 1.122 | 1.114 |
| 2012 | Wed | 1.109 | 1.139 |
| 2012 | Thu | 1.076 | 1.143 |
| 2012 | Fri | 1.057 | 1.117 |
| 2012 | Sat | 0.861 | 0.788 |
| 2012 | Holiday | 0.860 | 0.738 |
| 2012 | Non-Holiday | 1.140 | 1.127 |
| 2012 | Non-Long Holiday | 1.066 | 1.021 |
| 2012 | Long Holiday | 0.876 | 0.790 |
| 2013 | Sun | 0.773 | 0.670 |
| 2013 | Mon | 0.994 | 1.009 |
| 2013 | Tue | 1.122 | 1.115 |
| 2013 | Wed | 1.103 | 1.101 |
| 2013 | Thu | 1.067 | 1.146 |
| 2013 | Fri | 1.071 | 1.162 |
| 2013 | Sat | 0.869 | 0.793 |
| 2013 | Holiday | 0.862 | 0.749 |
| 2013 | Non-Holiday | 1.138 | 1.128 |
| 2013 | Non-Long Holiday | 1.066 | 1.026 |
| 2013 | Long Holiday | 0.878 | 0.791 |
| 2014 | Sun | 0.764 | 0.695 |
| 2014 | Mon | 1.017 | 1.045 |
| 2014 | Tue | 1.100 | 1.066 |
| 2014 | Wed | 1.095 | 1.105 |
| 2014 | Thu | 1.079 | 1.156 |
| 2014 | Fri | 1.076 | 1.131 |
| 2014 | Sat | 0.869 | 0.801 |
| 2014 | Holiday | 0.858 | 0.760 |
| 2014 | Non-Holiday | 1.142 | 1.122 |
| 2014 | Non-Long Holiday | 1.067 | 1.022 |
| 2014 | Long Holiday | 0.873 | 0.814 |
| 2015 | Sun | 0.768 | 0.681 |
| 2015 | Mon | 1.031 | 1.089 |
| 2015 | Tue | 1.128 | 1.123 |
| 2015 | Wed | 1.066 | 1.061 |
| 2015 | Thu | 1.069 | 1.124 |
| 2015 | Fri | 1.075 | 1.161 |
| 2015 | Sat | 0.863 | 0.757 |
| 2015 | Holiday | 0.858 | 0.737 |
| 2015 | Non-Holiday | 1.142 | 1.132 |
| 2015 | Non-Long Holiday | 1.063 | 1.017 |
| 2015 | Long Holiday | 0.881 | 0.818 |
| 2016 | Sun | 0.762 | 0.677 |
| 2016 | Mon | 1.040 | 1.131 |
| 2016 | Tue | 1.145 | 1.116 |
| 2016 | Wed | 1.106 | 1.096 |
| 2016 | Thu | 1.048 | 1.116 |
| 2016 | Fri | 1.049 | 1.101 |
| 2016 | Sat | 0.851 | 0.768 |
| 2016 | Holiday | 0.851 | 0.739 |
| 2016 | Non-Holiday | 1.149 | 1.126 |
| 2016 | Non-Long Holiday | 1.070 | 1.020 |
| 2016 | Long Holiday | 0.858 | 0.781 |
| 2017 | Sun | 0.752 | 0.678 |
| 2017 | Mon | 1.032 | 1.081 |
| 2017 | Tue | 1.142 | 1.118 |
| 2017 | Wed | 1.113 | 1.123 |
| 2017 | Thu | 1.068 | 1.121 |
| 2017 | Fri | 1.050 | 1.122 |
| 2017 | Sat | 0.843 | 0.764 |
| 2017 | Holiday | 0.847 | 0.742 |
| 2017 | Non-Holiday | 1.153 | 1.125 |
| 2017 | Non-Long Holiday | 1.071 | 1.017 |
| 2017 | Long Holiday | 0.870 | 0.825 |
| 2018 | Sun | 0.756 | 0.705 |
| 2018 | Mon | 0.999 | 1.016 |
| 2018 | Tue | 1.135 | 1.107 |
| 2018 | Wed | 1.117 | 1.069 |
| 2018 | Thu | 1.075 | 1.140 |
| 2018 | Fri | 1.065 | 1.147 |
| 2018 | Sat | 0.852 | 0.817 |
| 2018 | Holiday | 0.848 | 0.763 |
| 2018 | Non-Holiday | 1.152 | 1.118 |
| 2018 | Non-Long Holiday | 1.074 | 1.028 |
| 2018 | Long Holiday | 0.861 | 0.768 |
